# Editorial: Highlights in Autophagy—From Basic Mechanisms to Human Disorder Treatments

**DOI:** 10.3390/cells12010188

**Published:** 2023-01-03

**Authors:** Pei-Hui Lin, Lydie Combaret

**Affiliations:** 1 Davis Heart and Lung Research Institute, The Ohio State University, Columbus, OH 43210, USA; 2 Department of Surgery, The Ohio State University Wexner Medical Center, Columbus, OH 43210, USA; 3 Université Clermont Auvergne, INRAE, UNH UMR 1019, CRNH Auvergne, 63000 Clermont-Ferrand, France

## Introduction

Autophagy is an evolutionarily conserved catabolic process and represents a field of research that is constantly growing. Autophagy degrades cellular proteins, damaged/or excess organelles (ERphagy, ribophagy, mitophagy, pexophagy), protein aggregates (aggrephagy), lipids (lipophagy), or invading pathogens (xenophagy). Autophagy is also interconnected with the ubiquitination-proteasome system (UPS) and plays a major role in cell homeostasis, particularly for eliminating abnormal organelles, controlling the energy balance and proteostasis. Therefore, dysfunction of many forms of autophagy has been implicated in homeostasis maintenance and contributes to various diseases and aging processes. For a general overview on autophagy, precision medicine and emerging therapeutic goals, please refer to recent excellent review articles [1,2,3].

In this Research Topic, we collected seven articles to investigate in-depth molecular and cellular mechanisms that regulate autophagy in specific stress conditions and the role these processes play in pathophysiological conditions. 

Zhang and Chen reviewed the roles of plant NBR-1 (neighbor of BRCA1 gene 1), a homolog of the human selective autophagy cargo receptor SQSTM1/p62 family protein, acting in aggrephagy for the autophagic degradation of ubiquitinated proteins/substrates upon a broad spectrum of biotic and abiotic stresses such as heat tolerance and heat stress memory, salt, drought, oxidative stress, and plant anti-viral defense by targeting viral RNA granules [4]. Moreover, NBR1-mediated selective autophagy is involved in the regulation of the special protein secretion pathway, stress-induced pexophagy, nutrient responses, and plant hormone ABA signaling.

Hiebel et al. studied BAG3 chaperone complex-mediated selective autophagy in the regulation of the cellular proteostasis network via exploring the BAG3 proteomic signature during acute proteostasis stress (proteasome inhibition) [5]. The chaperone Hsp70 and its co-chaperone BAG3 (BCL-2-associated athanogene 3) have been shown to play critical physiological roles in the regulation of cellular proteostasis, stress responses, cell death, development, metabolism, and cytoskeletal dynamics. Mechanistically, BAG3-dependent signaling modulates apoptotic signaling and autophagic/lysosomal activity to remove misfolded protein. The authors identified novel potential BAG3 interactors and altered interactors upon stress that may elucidate a new functionality of BAG3 in disease models.

Ke provided a state-of-the-art overview on the role of mitophagy in the pathogenesis of liver diseases. Ke first detailed the molecular mechanisms of the regulation of autophagy, namely macroautophagy, microautophagy, and cargo receptor(s)-dependent selective autophagy, which mainly occurs during the quality control of organelles [6]. Particular attention was paid to the role of hepatic mitophagy in the regulation of liver physiology, such as glycogen metabolism, turnover of nutrients and metabolites, and homeostasis of macromolecules (RNA, proteins, etc.) and organelles. Additionally, the literature supporting the dysfunction of mitophagy in association with various liver diseases including hepatocellular carcinoma, injury, viral hepatitis, steatohepatitis, fatty livers, and cancers was reviewed.

Shi et al. demonstrated that a high-phosphate diet (phosphotoxicity) contributed to the interplay of autophagy and apoptosis in cardiomyopathy [7]. Wildtype mice fed with high dietary phosphate loading caused cardiac hypertrophy and fibrosis. Interestingly, the high-phosphate diet exacerbated Atg-5 deficiency-induced pathologic cardiac remodeling through a parallel autophagy-independent yet apoptosis-inductive mechanism in cardiomyocytes. Therefore, enhancing autophagy and/or the control of phosphate levels could be potential therapeutic strategies for metabolic cardiomyopathy.

Griffin et al. identified the roles of hepatic lipid droplet (LD)-associated Perilipin2 (Plin2) at the intersection of the modulation of classical lipolysis (through adipose triglyceride lipase (ATGL)) and autophagic lipolysis (lipophagy) in hepatic LD-lipids metabolism and energy homeostasis [8]. An imbalance of storage or the breakdown of LD-lipids and an aberrantly high Plin2 level have been associated with hepatic steatosis in non-alcoholic fatty liver disease (NAFLD). In this study, liver-specific Plin2 deficiency mice were protected from diet-induced NAFLD through integrated protective mechanisms of both enhanced autophagy and ATGL-mediated lipolysis to reduce hepatic triglyceride accumulation within LD, which also simultaneously increased Fatty Acid Oxidation (FAO). The implication of manipulating Plin2 as a potential therapy to treat other diseases such as insulin resistance and obesity was also discussed.

Lypova et al. evaluated PFKFB3, a critical kinase in glycolysis, and its involvement in autophagy-associated chemoresistance toward non-small cell lung carcinomas (NSCLCs). Erlotinib is a tyrosine-kinase-inhibitor (TKI) targeting EGFR and is often used in the treatment of active EGFR (mutations)-driven NSCLCs [9]. However, the clinical outcome of Erlotinib is hampered by chemoresistance, so an effect might be linked to prosurvival autophagy within cells. Using combinational pharmacological PFKFB3 inhibitors and/or autophagic inhibitors, the authors identified that PFKFB3 is a novel mediator of erlotinib-induced cytoprotective autophagy in NSCLCs. Collectively, the addition of the PFKFB3 inhibitor provided dual benefits on the impairment of erlotinib-induced autophagy and improvement of the cytotoxicity of Erlotinib, thus suggesting a new strategy against drug-resistant NSCLCs.

Wang et al. investigated a current phase III clinical trial chemical, “Lupuzor/P140”, a 21 a.a phosphopeptide epitope derived from 70K-splicesomal protein U1 small nuclear RNP that was first identified in lupus-prone mice; its molecular mechanism was investigated as a treatment of lupus [10]. Lupus is a complex and often heterogenous autoimmune disease that involves major components of the immune system manifested by hyper-inflammation. Dysfunctional autophagy and potential risk genetic loci on autophagy have been proposed to be associated with lupus. The authors demonstrated the lysosomal malfunction in lupus splenocytes. Moreover, this showed P140 entering hepatic lysosome and inhibiting chaperone-mediated autophagy (CMA) hyperactivity through hampering the CMA substrates lysosomal uptake in lupus MRL/lpr mice. Therefore, this study suggested P140 possibly exerts an immunomodulatory protective function to remodel the autophagy-lysosomal pathway in lymphocytes from lupus-prone mice and that it could have broad applications to other autoimmune conditions.

In conclusion, this Research Topic initiates the discussion of how autophagy impacts on cellular physiology in the modulation and pathogenesis of various disease states. The emerging precision medicines that could improve or restore compromised autophagy are now actively investigated and expected to be tested in clinical trials to benefit susceptible individuals [2]. Lastly, I would like to deeply thank all the authors for their valuable contributions to this Special Issue.
